# Anesthetic Efficacy of Endo-Ice and Intrapulpal Ice Sticks After Inferior Alveolar Nerve Block in Symptomatic Irreversible Pulpitis: A Randomized Controlled Study

**DOI:** 10.7759/cureus.42135

**Published:** 2023-07-19

**Authors:** Rethi Gopakumar, Mahesh Jayachandran, Sreelekshmi Varada, Jitha Jayaraj, Jenaki Ezhuthachan Veettil, Naveen S Nair

**Affiliations:** 1 Department of Conservative Dentistry and Endodontics, Noorul Islam College of Dental Sciences, Trivandrum, IND; 2 Department of Periodontics, Noorul Islam College of Dental Sciences, Trivandrum, IND; 3 Department of Conservative Dentistry and Endodontics, Sri Sankara Dental College, Trivandrum, IND; 4 Department of Conservative Dentistry and Endodontics, PSM College of Dental Science and Research, Thrissur, IND; 5 Department of Conservative Dentistry and Endodontics, Vydehi Institute of Dental Sciences and Research Centre, Bangalore, IND

**Keywords:** heft-parker visual analog scale, intrapulpal ice sticks, inferior alveolar nerve block, endo-ice, dental anxiety, cryotherapy

## Abstract

Background: There is a dearth of studies to assess the effectiveness of cryotherapy as a possible adjuvant in anesthesia. Therefore, this study aimed to compare the impact of pretreatment intraoral ice packs, application of Endo-ice, and a combination of Endo-ice and intrapulpal ice sticks use on the effectiveness of pulpal anesthesia as an adjunct in the management of pain and dental anxiety in mandibular second molars with symptomatic irreversible pulpitis (SIP) against traditional inferior alveolar nerve block (IANB) technique.

Materials and methods: The randomized controlled double-blind clinical study comprised of 200 subjects within the age group of 20-45 years with SIP involving mandibular second molars and divided into four groups of 50 each. Conventional IANB was administered in group 1. Intraoral compact ice packs, Endo-ice, and combined use of Endo-ice and intrapulpal ice sticks were employed following conventional IANB in groups 2, 3, and 4, respectively. The intensity of pain and anxiety before and after the intervention was documented using Heft-Parker visual analog scale (HP-VAS) and Corah's Dental Anxiety Scale-Revised (DAS-R).

Results: The average age of the overall study sample was 30.8±2.08 years, and the differences in age and gender distribution were found to be statistically insignificant. The mean HP-VAS scores on access opening and pulpectomy, using ANOVA paired with Tukey's post hoc multiple comparison tests were evaluated to be statistically highly significant (p<0.001). The greatest reduction in HP-VAS score was observed in group 4 receiving combined use of both Endo-ice and intrapulpal ice sticks. While the pretreatment DAS-R score was determined to be statistically insignificant between groups, group 1 subjects had the highest DAS-R score postoperatively (p<0.001). The effectiveness of pulpal anesthesia was found to be 84%, 96%, 92%, and 98% for groups 1 through 4, respectively, and demonstrated a statistically significant difference.

Conclusion: The combined use of Endo-ice and intrapulpal ice sticks used as an adjuvant to local anesthesia (LA) was found to be significantly effective in lowering pain compared to the control groups in molars with SIP. Cold administration before and during the procedure was more effective than conventional LA in lowering intraoperative anxiety. Furthermore, the use of Endo-ice and intrapulpal cold after IANB significantly improves the effectiveness of pulpal anesthesia in mandibular second molars with SIP.

## Introduction

The inflamed pulp in symptomatic irreversible pulpitis (SIP) causes acute pain that may either be spontaneous, or induced by heat stimuli and can last for 30 seconds or even further following the removal of stimulus, establishing the necessity for endodontic therapy. It is critical to accomplish effective pulpal anesthesia during root canal therapy of molars with SIP for pain control and anxiety management. It is particularly challenging for individuals with SIP in mandibular molars as the inferior alveolar nerve block (IANB) typically fails to deliver substantial anesthesia [1]. Clinically, several adjunct anesthetic approaches have been employed to manage a tooth that defies anesthesia, and the efficacy rates of obtaining pulpal anesthesia with these strategies vary. It has been noted that 5-10% of patients are likely to need intrapulpal anesthesia for the continuation of the endodontic procedure. The success of intrapulpal anesthesia has been attributed to the backpressure induced during local anesthesia (LA) administration, regardless of the LA agent being administered. However, the patient will experience extreme discomfort while the intrapulpal anesthetic is being delivered [2].

Several clinical investigations were conducted to address the challenges of IANB in patients with SIP. These researches evaluated various LA solutions to lidocaine, which presently constitutes the most often administered anesthetic in dentistry and can be regarded as the benchmark [3]. Preoperative intraoral cryotherapy enhanced the efficacy of IANB in SIP patients by delivering LA by decreasing the activation thresholds of nociceptors and pain signal transmission rates, presenting brief analgesia, and reducing blood circulation, metabolic rate, and cell-based oxygen requirements [4,5]. In teeth with SIP, cooled 2% lignocaine hydrochloride with 1:100,000 epinephrine served as a viable alternative for standard anesthetic for attaining a successful outcome, lesser pain, and more rapid onset during the process of endodontic therapy [6]. Cryotherapy entails decreasing tissue temperature to manage the disease and reduce inflammation. It has applications in dentistry to alleviate edema and pain following surgery or impactions to relieve postoperative endodontic discomfort [7-10].

Not many investigations have been conducted to assess the effectiveness of cryotherapy as a possible adjuvant in anesthesia [11]. It has been demonstrated that using intraoral cryotherapy using compact ice sticks increases the efficacy of IANB in mandibular molars with SIP [12]. Endo-ice and ice sticks are also effective as adjuvant to IANB in lowering discomfort during pulpectomy [13]. The use of intrapulpal ice sticks is justified by the idea of thermal stimulation and the physiological reaction of the tooth pulp to temperature fluctuations. Cold may also be used as an additional anesthetic approach to lessen endodontic discomfort during intraoperative procedures. When given to the tooth, cold in the form of Endo-ice and ice sticks may assist to lessen the discomfort experienced during pulp extirpation.

Therefore, the study aimed to compare the impact of preoperative intraoral small ice packs, application of Endo-ice, and a combination of the use of Endo-ice and intrapulpal ice sticks on the effectiveness of pulpal anesthesia as an adjunct in the management of pain and dental anxiety in mandibular second molars with SIP against conventional IANB technique.

## Materials and methods

**Figure 1:**
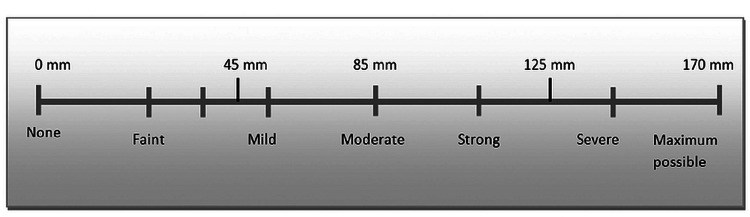
Heft-Parker visual analog scale.

This double-blind randomized controlled clinical study was designed following the Declaration of Helsinki and in compliance with the consolidated standards of reporting trials statement [14]. The institutional ethics committee approved the study (#IEC/006/2016). Following a preliminary evaluation of 258 patients, the study enrolled 200 patients who satisfied the inclusion criteria. After explaining the purpose of the study, its objectives, methods, anticipated benefits, and risks, all the study subjects gave their voluntary informed consent. Baseline variables such as age, gender, pretreatment pain, and scoring of dental anxiety were documented. G-power software (Düsseldorf, Germany: Heinrich Heine University Düsseldorf) (version 3.1.9.7) was utilized to estimate the sample size of 50 per group at a 5% significance level, 80% statistical power, with an effect size of 0.25, and a projected attrition rate of 10% in each group. The degree of pain before and after the intervention was recorded by employing a 170-mm Heft-Parker visual analog scale (HP-VAS) (Figure 1) [15].

Before administering LA, each patient was provided with an extensive overview of the HP-VAS, which was categorized into four distinct groups: a lack of pain referred to 0 mm, mild pain was characterized as a score exceeding 0 mm but not greater than 54 mm and encompassed the characterizations of faint, weak, and mild pain. Moderate pain was outlined as a score above 54 mm and lower than or equivalent to 114 mm and comprised the categories of strong, intense, and maximum possible pain. The dental anxiety was assessed before and after intervention by employing Corah's Dental Anxiety Scale-Revised (DAS-R), with an overall score between one and 20. A numerical rating of below nine suggests mild anxiety. A score of 9-12 indicates moderate anxiety, 13-14 suggests high anxiety, and 15-20 implies phobia/severe anxiety [16].

Patients within the age group of 20-45 years with SIP and scheduled for root canal therapy in mandibular second molars were assessed for the following factors to be included in the study: a pretreatment HP-VAS score of above 54 suggesting moderate to severe pain, DAS-R score greater than nine, a positive response to electric pulp testing (EPT), and an extended response to cold test demonstrating pulp sensitivity. SIP was diagnosed for each tooth upon reviewing clinical and radiographic records. The exclusion criteria include those patients under the age of 19 years who disclosed pain on cold application, non-vital teeth, calcified root canals, teeth with periapical radiolucency or abscess with/without sinus opening, teeth with periodontitis, individuals with systemic disease, or with a history of previous endodontic treatment, allergies, or antibiotics intake in the preceding 48 hours.

A digitally-generated randomization process was used to allocate recruited individuals to one of the four groups under study. The principal investigator and the individuals being studied were both blinded to the groups they were assigned to. The randomized sequence of numbers was made with unique codes for particular individuals and was hidden in consecutively assigned opaque, individualized confidential packages, which were unsealed by the clinician during treatment. The investigator was an endodontist with over 10 years of expertise. Age, gender, preoperative discomfort, and anxiety were documented and the entire procedure was standardized by using the same operator, materials, technique, and clinical conditions.

Group 1 (negative control group)

Conventional IANB was delivered employing a 27-gauge needle of 31 mm with 2 mL of 2% lignocaine hydrochloride with adrenaline 1:80000. The tip of the needle was placed slightly laterally in the center of the pterygomandibular raphe to approach the bone, gently retracted, and aspirated. The solution was delivered at an average rate of one mL/minute. The effectiveness of anesthesia was established by utilizing EPT after 15 minutes with two successive negative responses to a reading of 80 µA. The molars were isolated using a rubber dam and an access opening was carried out.

Group 2 (positive controls)

A conventional IANB was delivered as in group 1 following which small cold packs packed in sterile gauze were applied intraorally on the vestibular region. The study subjects were advised to maintain the ice packs for 5 minutes and take them out for a moment if they felt excessive cold or discomfort.

Group 3 (Endo-ice group)

The typical IANB was given as indicated in group 1. Endo-ice (1,1,1,2 tetrafluoroethane) was subsequently applied for a total of 10 seconds on the buccal, lingual surfaces (3 seconds each), and for 4 seconds on the occlusal surfaces preceding access opening.

Group 4 (Endo-ice and ice sticks group)

**Figure 2:**
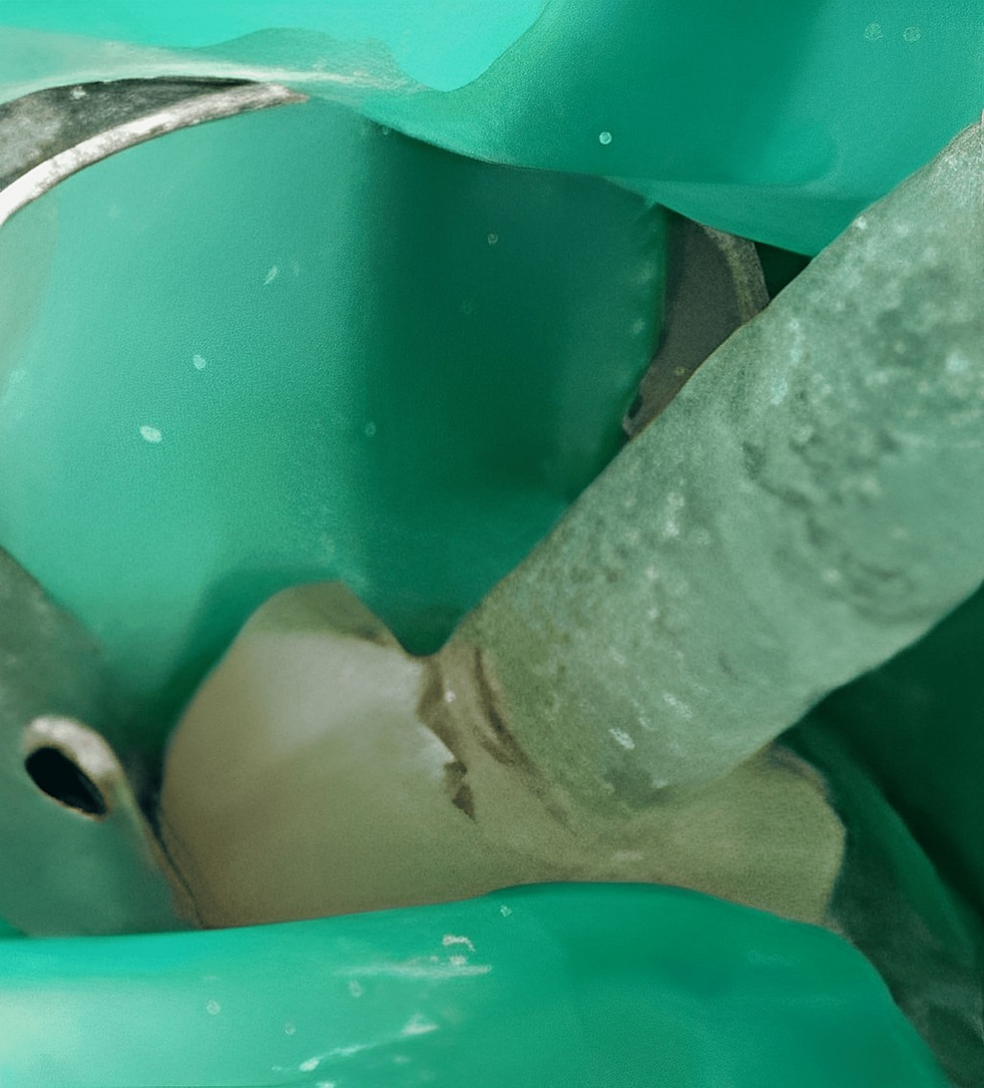
Cold ice sticks used in the pulp chamber.

Endo-ice and intrapulpal ice sticks were employed as cold application methods. IANB was delivered as previously stated. Endo-ice was then applied as stated in group 3 before access opening followed by deroofing and locating the canals. Subsequently, two ice sticks were placed directly in the pulp chamber for 4 minutes (Figure 2).

Each ice sticks were with a diameter and length of 8 mm and 2 cm, respectively. The success of anesthesia was established after 15 minutes, as mentioned in group 1. The HP-VAS and DAS-R scales were used to assess the pretreatment pain and dental anxiety. A pretest comprising 20 patients was performed to determine the internal coherence of the questionnaires, exhibiting Cronbach's alpha of 0.84. Prior to the IANB, all groups received a 60-second application of 20% benzocaine anesthetic gel at the injection site. The endodontic therapy was commenced after evaluating lip numbness and two consecutive negative responses to the EPT following maximum activation. Those with positive responses had been excluded and received intraligamentary and/or intrapulpal injections. A rubber dam was used to isolate the teeth followed by an access opening. During the treatment procedure, individuals were prompted to rate their pain and anxiety at three different points, such as pre-treatment, during access opening, and immediately following pulpectomy.

The canal length was determined using an apex locator (ProPex Pixi; Charlotte, NC: Dentsply). The periapical radiographs were used to ascertain the operating length. Nickel-titanium rotary files (ProTaper Universal; Charlotte, NC: Dentsply) were used for instrumentation of the canal using K-files up to #15 size. With the determined working length, the ideal size of the apical preparation has been estimated to be three sizes bigger than the initial file binding. During the process of instrumentation, each canal received one-minute irrigation using 2 mL of 17% EDTA with intermittent irrigation using 5 mL of distilled water. Obturation was performed with gutta-percha and interim restoration was placed. The endodontic therapy concluded a week thereafter.

Statistical analysis

The IBM SPSS Statistics version 26.0 for Windows (Armonk, NY: IBM Corp.) was utilized for statistical analysis. Normal probability plots were used to check normality of data. Data were expressed as mean±SD and percentages for quantitative and categorical variables, respectively. The age, HP-VAS, and DAS-R score differences across groups were evaluated using one-way ANOVA paired with Tukey's post hoc tests for multiple comparisons. The chi-square test was applied to analyze the differences in gender and LA success rate between the groups.

## Results

In this study, 200 subjects with SIP in mandibular second molars were assigned to one of four groups randomly, each with 50 subjects receiving conventional LA (group 1), intraoral ice packs (group 2), Endo-ice (group 3), or a combination of Endo-ice and intrapulpal ice sticks (group 4). The mean age of the overall study sample was 30.8±2.08 years, and the differences in age distribution across the groups were reported to be statistically insignificant (p=0.5). The study included 102 males and 98 females, and the differences in gender distribution between study groups were also statistically insignificant with p=0.94 (Table 1).

**Table 1:** Frequency of age and gender among the study sample.

Variables		Group 1 (n=50)	Group 2 (n=50)	Group 3 (n=50)	Group 4 (n=50)	p-Value
Age (years)	Mean±SD	30.4±2.1	31±2.08	30.8±2.11	30.9±2.04	0.50
Gender	Male	24	25	27	26	0.94
	Female	26	25	23	24	

Applying one-way ANOVA, the mean difference in pretreatment HP-VAS scores across the groups was determined to be statistically insignificant (p=0.38). The mean HP-VAS scores on access opening and pulpectomy, on the other hand, were determined to be statistically highly significant (p<0.001). Tukey's post hoc multiple comparison tests demonstrated statistically highly significant differences between negative controls, positive controls, and the use of combined Endo-ice and intrapulpal ice sticks (groups 1 and 2, and groups 1 and 4) during access opening. Similarly, statistically highly significant differences during access opening were found between groups 2 and 4 and groups 3 and 4. During pulpectomy, the intergroup comparisons of HP-VAS score were shown to exhibit statistically significant differences across the groups, except for groups 2 and 3. The greatest reduction in HP-VAS score was observed in group 4, which received combined use of both Endo-ice and intrapulpal ice sticks during access opening and pulpectomy procedures. During access opening and pulpectomy, the HP-VAS scores for groups 2 and 3 were observed to be roughly comparable (Table 2).

**Table 2:** The mean (SD) of HP-VAS score at different treatment phases among the study sample utilizing one-way ANOVA and Tukey's post hoc tests. *Statistically highly significant. **Statistically significant. NS: not significant; HP-VAS: Heft-Parker visual analogue scale

Variables						Tukey's post hoc test					
Treatment phases	Group 1	Group 2	Group 3	Group 4	p-Value	1↔2	1↔3	1↔4	2↔3	2↔4	3↔4
Pretreatment	113.28 (2.53)	112.5 (2.12)	113.07 (2.46)	112.88 (2.12)	0.38	NS	NS	NS	NS	NS	NS
Access opening	53.73 (1.93)	52.47 (2.18)	52.98 (2.2)	50.83 (1.91)	<0.001*	*	NS	*	NS	*	*
Pulpectomy	45.44 (3.69)	42.97 (2.81)	42.07 (2.69)	41.06 (2.21)	<0.001*	*	*	*	NS	*	**

The pretreatment DAS-R score was determined to be statistically insignificant between groups, indicating that the study subjects experienced similar degrees of anxiety before treatment (Table 3). However, group 1 subjects had the highest DAS-R score postoperatively, and mean differences across groups 1 and 2, groups 1 and 3, groups 1 and 4, groups 2 and 4, and groups 3 and 4 were revealed to be statistically significant. The effectiveness of pulpal anesthesia was found to be 84%, 96%, 92%, and 98% for groups 1 through 4, respectively. The chi-square test demonstrated a significant difference in the effectiveness of IANB across the groups as elicited using EPT (Table 4).

**Table 3:** The mean (SD) of DAS-R score at different treatment phases among the study sample utilizing one-way ANOVA and Tukey's post hoc tests. *Statistically highly significant. *Statistically significant. NS: not significant; DAS-R: Dental Anxiety Scale-Revised

Variables						Tukey's post hoc test					
Treatment phases	Group 1	Group 2	Group 3	Group 4	p-Value	1↔2	1↔3	1↔4	2↔3	2↔4	3↔4
Pretreatment	11.77 (2.49)	11.85 (2.28)	12.03 (2.9)	12.17 (2.27)	0.85	NS	NS	NS	NS	NS	NS
Postoperative	10.92 (3.05)	9.44 (2.54)	9.07 (2.72)	8.01 (2.62)	<0.001*	*	*	*	NS	*	**

**Table 4:** Responses to electric pulp testing following IANB. IANB: inferior alveolar nerve block

Electric pulp testing	Group 1, n (%)	Group 2, n (%)	Group 3, n (%)	Group 4, n (%)	Total, n (%)	p-Value
Positive response	8 (16%)	2 (4%)	4 (8%)	1 (2%)	10 (5%)	0.04
Negative response	42 (84%)	48 (96%)	46 (92%)	49 (98%)	190 (95%)	
Total	50 (100%)	50 (100%)	50 (100%)	50 (100%)	200 (100%)	

## Discussion

This study assessed the adjunct techniques necessary for successful IANB in mandibular second molars with SIP. Age, gender, preoperative discomfort, and anxiety levels were comparable between groups. In mandibular molars with SIP, the effectiveness of IANB varies between 15% and 57%. It is crucial to consider that effective anesthesia of the IANB and associated tissues might be exhausting due to anatomical variances, inflammation, the technique employed, needle deflection, competence of the operator, and the patient's dental fear [12]. Furthermore, proper pain management could substantially lessen the anxiety experienced during endodontic therapy [17].

Analgesic effects are felt when cryotherapy is administered to the intended locations. Cryotherapy also delays brain signals and inhibits the production of chemical mediators involved in pain transmission by reducing the conduction rate of pain impulses [12,18]. These effects may account for the greater effectiveness of IANB among the study groups. Goodis et al. revealed that cooling the teeth lowered pulpal blood supply and in certain instances, eliminated the sense of pain [19]. De Morais et al. investigated the decline in temperature of the coronal pulp of incisors subjected to various refrigerated sprays and also observed that applying Endo-ice to the tooth surface for 10 seconds culminated in a decline in the pulpal temperature of 9.6°C [20]. Apparently, a greater amount of thickness of the residual dentin of molars might have resulted in a lowering of heat conductivity of dentin in the present study, leading to inadequate cooling of Endo-ice, which was in agreement with the prior findings [13].

The analgesic impact of cold application can be enhanced by prolonging the duration of cryotherapy. However, a tooth being subjected to excessive hot or cold temperature has been shown to induce thermal fatigue defects [13]. According to Vera et al., the ideal cryotherapy time required is determined by the characteristics of the soft tissues around it. When the thickness of the soft tissues is lesser, a 5-minute cryotherapy session is satisfactory [21]. The current investigation adopted this guideline to avert probable tissue damage, which was consistent with a previous study [12]. There were no adverse effects reported in the area where the cold application was used in any of the study subjects.

The success of IANB is frequently judged by the objective and subjective manifestations reported. Nevertheless, an EPT is a more reliable technique for evaluating pulpal anesthesia. A lack of response to EPT is the hallmark of profound pulpal anesthesia [22]. The HP-VAS utilized in the study is simple to accomplish the subjective method of quantifying pain that has been deployed in many prior studies to appraise intraoperative pain [12,23,24]. Dental anxiety is a significant element that can influence the pain perception of an individual, as anxious patients have a lower pain threshold. In our investigation, the DAS-R was used to assess dental anxiety in all individuals both prior to and following the intervention, alongside the pain scale. The preoperative state of anxiety was standardized by selecting those with moderate anxiety. The postoperative DAS-R scores were considerably lower in the cold group in contrast to the controls, which could be attributed to enhanced anesthesia of cold application.

According to a recent systematic review, while intracanal cryotherapy is a simple and affordable treatment for reducing postoperative endodontic discomfort, the degree of reliability of evidence demonstrated in the literature is inadequate [7]. Furthermore, varying pain thresholds across patients may influence HP-VAS scores [12]. Further study is warranted to evaluate the impact of cold against various additional anesthetic approaches, such as intraligamentary and intraosseous injections, along with other LA agents.

## Conclusions

The study findings showed that the combined use of Endo-ice and intrapulpal ice sticks used as an adjuvant to LA was significantly effective in lowering pain during endodontic therapy compared to the control groups. Cold administration before and during the procedure was more effective than conventional LA in lowering intraoperative anxiety. Furthermore, the use of Endo-ice and intrapulpal cold after IANB significantly improves the effectiveness of profound pulpal anesthesia in patients with SIP involving mandibular second molars. Additionally, more intensive studies in clinical settings are critical to evaluate the potential merits of this low-cost technique for routine therapeutic application in conditions where pain and inflammation are predicted.
